# CIP2A overexpression induces autoimmune response and enhances JNK signaling pathway in human lung cancer

**DOI:** 10.1186/s12885-015-1899-0

**Published:** 2015-11-11

**Authors:** Bo Peng, Yurong Chai, Yang Li, Xinxin Liu, Jianying Zhang

**Affiliations:** Border Biomedical Research Center & Department of Biological Sciences, The University of Texas at El Paso, El Paso, TX 79968 USA

**Keywords:** Tumor-associated antigen, Cancerous inhibitor of PP2A, c-Jun activated kinase (JNK), Mitogen activated kinase 4 (MKK4), Cell proliferation

## Abstract

**Background:**

Cancerous inhibitor of PP2A (CIP2A) is a recently characterized oncoprotein, which promotes cancer cell proliferation. But the role of CIP2A in lung cancer progression is still not well understood.

**Methods:**

The expression level of CIP2A in lung cancer tissues was examined by immunohistochemistry. CIP2A-associated cell proliferation was performed by knock down or overexpression of CIP2A in lung cancer cells. Phospho-array was used to screen kinase candidates related to expression change of CIP2A. Western-blot and luciferase reporter assay were used to validate phospho-array results.

**Results:**

Overexpression of CIP2A in lung cancer not only triggers immune response in lung cancer patients but also promotes lung cancer cell proliferation. By phospho-array, several kinase candidates were identified, one of which is c-Jun activated kinases (JNK). The knock down of CIP2A decreased JNK phosphorylation, and the phosphorylation of downstream transcriptional factors, ATF2 and c-Jun, whose transcriptional activity were decreased as well. Furthermore, the expression level of CIP2A also affected the phosphorylation of the upstream kinase of JNK, MKK4/MKK7. At last, treatment with JNK inhibitor partially abolished CIP2A-induced cell proliferation.

**Conclusion:**

CIP2A is a tumor-associated autoantigen in lung cancer, which promote lung cancer proliferation partially through MKK4/7-JNK signaling pathway.

## Background

Lung cancer is one of the major causes of cancer-related deaths both in men and women throughout the world. The 5 year survival rate of lung cancer is as low as 15 % [1]. Detection of lung cancer at early stage allows the patients to receive appropriate treatment with more options and less invasive surgery, thus decreasing the mortality and suffering of patients [2, 3]. However, the disease-associated symptoms imposed a challenging situation for early cancer diagnosis. Those symptoms can be hardly felt by the patients themselves until the disease is progressed to an advanced stage, which makes lung cancer diagnosed at a late stage when the cancer cells have metastasized [2]. Although chest x-ray check and spiral CT scans have been used in routine screening of lung cancer, they are ineffective methods with insufficient accuracy due to the limited technology capabilities, observer variations and data interpretations. Thus, the development of reliable biomarkers for early detection of lung cancer is an urgent field since there is no currently approved screening test for lung cancer detection.

Blood proteins have always been attractive targets for biomarkers in many different types of diseases including cancer. The expression change of blood proteins is associated with the progression of diseases [4, 5]. Autoantibodies are antigen-driven responses that can be viewed as reporters from the immune system. The identity of antigens suggested antigens sensed by the immune system might play roles in the pathophysiology of the disease process [5, 6]. In cancer patients, most of the autoantigens targeted by host’s immune system were identified as proteins in cell transformation and cancer progression e.g. mutant p53, K-ras, insulin-growth factor II mRNA binding protein family (IMPs), c-myc, p90/CIP2A, p16, p21, cyclin A, B and E [6, 7]. Thus the identification of novel tumor-associated antigens may provide insights into proteins whose aberrant expression can be sensed by the immune system, and these cancer-associated antigens or antibodies may also be used as biomarker candidates for the development of non-invasive cancer detection method.

CIP2A was initially identified as a cancer-associated autoantigen in gastric and liver cancer. But the function of CIP2A was largely unknown due to the lack of homology of CIP2A to any other proteins [8]. Until 2007, CIP2A was characterized as a cognate interactor of protein phosphatase 2A (PP2A). Functional analysis of the CIP2A by genetic, biochemical and animal studies revealed that CIP2A can selectively inhibit the phosphatase activity of certain PP2A complexes, stabilizing one of the PP2A targets, c-myc without affecting other PP2A substrates like mouse double minute 2 oncoprotein (MDM2). Although following studies showed that the expression of CIP2A was up-regulated in a variety of cancer types ranging from solid tumor to hematological malignancies, research on the cellular functions of CIP2A under physiological conditions and in diseases states are still limited [9–16] . Only few CIP2A substrates had been identified so far including two proteins, protein kinase B (PKB/AKT) and UNCEB5. Depletion of CIP2A via siRNA in hepatocellular carcinoma, breast cancer and esophageal cancer not only decreased cancer cell proliferation but also sensitized cancer cells to chemotherapeutical agents like bortezomib and tumor necrosis factor. One of the mechanisms was the contribution of CIP2A to enhance AKT-mediated cell survival through the direct regulation of AKT-associated PP2A phosphatase activity. In addition, the expression of CIP2A in endothelial cells promotes cell survival under ligand-dependent cell signaling [17, 18]. The latest finding of CIP2A in controlling cell-cycle progression dramatically advanced our understanding of CIP2A in cancer progression. Two independent groups individually found CIP2A directly bind to cell-cycle regulators including polo-like kinase (PlK1) and never in mitosis gene A-related kinase 2 (NEK2) [19, 20]. CIP2A is able to translocate into the nucleus in mitotic entry, where it binds to Plk1 and became deposited in spindle pole [19]. In addition, CIP2A was also found in centrosome at G2/M stage. Both of these studies showed that the depletion of CIP2A resulted in the extended cell division [20]. These results implied that CIP2A has a broader role in regulating cell proliferation as previously expected [21]. Therefore, more functional roles of CIP2A in cancer progression need to be explored.

During the search of novel tumor-associated autoantigens in lung cancer patients, we found that CIP2A was overexpressed in lung cancer and elicited autoimmune response in lung cancer patients. The depletion and overexpression of CIP2A via genetic methods decreased and increased cancer cell proliferation, respectively. To define the function of CIP2A in cell proliferation, phospho-array containing key phosphorylation molecules in cancer was used. Our studies showed that CIP2A positively regulates the phosphorylation of AKT, p38 kinase and c-Jun activated kinase (JNK). The further analysis of the JNK pathway showed that CIP2A is a positive regulator of mitogen-activated kinase 4 (MKK4)-JNK signaling pathway in response to growth factors. Inhibition of JNK activation by JNK inhibitor partially abolished CIP2A-induced cell proliferation. Therefore, our study proposed a novel function of CIP2A in cancer progression.

## Methods

### Cell culture

Non-small cell lung cancer cell lines, A549, NCI-H838, NCI-H1299 and NCI-H460 and two small cell lung cancer cell lines, H69 and H146 were purchased from Amercian tissue collection center (ATCC). All of the cell lines, except A549, were cultured in RPMI-1640 medium (Invitrogen, Carlsbad, CA) containing 10 % fetal bovine serum (FBS) (Invitrogen) at 37 °C with 5 % CO_2_. A549 is cultured with the same conditions as for other cell lines, except that it is cultured in F12K medium (Invitrogen). The immortalized cell line BEAS-2B was purchased from ATCC and was cultured in LHC-9 medium (Invitrogen). HBEC3 cell was generally provided by Dr. Jerry W. Shay (University of Texas Southwestern Medical Center). This cell line was cultured in Keratinocyte-SFM supplemented with 50 μg/mL bovine pituitary extract and 5 ng/mL epidermal growth factor (Gibco) at 37 °C on porcine gelatin-coated tissue dishes as previously described [22].

### Serum sample

In this study, sera from 207 patients with lung cancer and 82 normal human sera were obtained from the serum bank in the investigator’s (Zhang) laboratory at The University of Texas at El Paso (UTEP), U.S.A. Normal human sera were collected from adult individuals who had no obvious evidence of lung diseases during annual health examinations. All lung cancer patients were diagnosed according to the criteria described in a previous study [23]. Informed consent was obtained from all the participants, including patients and normal human beings, by our clinical collaborators. This study was approved by the Ethic Committee of The University of Texas at El Paso: Institutional Review Board of The University of Texas at El Paso.

### Purification of autoantibodies from sera

To purify CIP2A-specific autoantibody from human sera, A549 total cell lysates were separated on 12 % SDS-PAGE gel and then transferred onto nitrocellulose membrane. The membrane around 90 kDa was cut off from the whole membrane, and then further cut into small pieces. Those pieces of membrane were incubated with individual serum containing autoantibody against 90 kDa autoantigen for 2 h at RT. The membranes were subsequently washed three times with TBST (10 min per washing cycle). Autoantibodies bound on the membrane were eluted with 1 M glycine solution, pH 3.0, which was immediately neutralized with 1 M Tris–HCl, pH 8.0 [24].

### ELISA

Recombinant GST-CIP2A fusion protein was purified using GST glutathione beads. The protein purity was >95 % by SDS-PAGE. Proteins were diluted in PBS to a final concentration of 0.5 μg/mL for coating polystyrene 96-well microtiter plates (Dynatech Laboratories, Alexandria, VA). A volume of 200 μL of each human serum samples at 1:200 dilutions was added to the antigen-coated wells and incubated for 1.5 h at RT. Horseradish peroxidase-conjugated goat antihuman IgG (Caltag Laboratories, San Francisco, CA) at 1:5,000 dilution and the substrate 2,2’-azinobis (3-ethylbenzthiazoline-6-sulfonic acid) (Boehringer Mannheim GmbH, Mannheim, Germany) were used as detecting reagents. Each sample was tested in duplicate, and the average OD at 405 nm was used for data analysis. The cutoff value designating positive reaction was the mean optical density (OD) of 82 normal human sera plus 3 standard deviations (SD). The detailed protocol of ELISA was used as described.

### Tumor tissue array, H&E staining and immunohistochemistry (IHC)

Formalin fixed and paraffin embedded lung cancer tissue arrays and normal human lung tissue were obtained from *Cybrdi* (Rockville, MD). The lung cancer tissue array contained 71 individual cases and 72 cores. Each of the cores represents one donor. The array contained normal human lung tissues from 23 individuals, spotted in triplicates. For both of these two tissue slides, each array spot was 1.5 mm in diameter and 5 μm in thickness. All the slides were stained with H&E staining and verified by pathologists. IHC was performed according to manufacturer’s protocol. Tumor tissue arrays were deparaffinized in Xylene, followed by rehydration in alcohol and then boiled in citrate buffer for antigen retrieval. Before antibody incubation, tissue array slides were incubated with hydrogen peroxide to block any endogenous peroxide activity and normal mouse antibody to reduce the nonspecific binding. The mouse monoclonal CIP2A antibody was added to the slides at a concentration of 1:50 and incubated at room temperature for 1 h, and goat anti-mouse HRP was used as secondary antibody. After washing, the peroxides reaction was developed with 3,3’-diaminobenzidine (DAB). At last, the slides were covered with cover slides mounted with mounting media.

Both of the slides were examined by two independent, blinded investigators. Five views were examined per slide, and 100 cells were observed per view at 400× magnification. IHC staining of CIP2A was scored by following a semi quantitative scale (− to +++), which evaluated the intensity in tumor areas, and the percentage of cells showing significantly higher immunostaining than control cells in normal lung tissues. Nuclear and/or cytoplasmic immunostaining in tumor cells was considered positive staining. The intensity of CIP2A staining was scored as 1 (mild), 2 (weak), and 3 (strong). Percentage scores were assigned as 0, 0 %; 1, 1–50 % and 2, 50–100 %. The scores of each tumor sample were multiplied to give a final score of 0–6, and the tumors were finally determined as lower expression, score 0–3 and high expression, score 4–6.

### Western-blot analysis

Cells were plated in 6-well tissue culture plates at 80 % confluence and incubated overnight. Cell lysates were obtained from transduced cells using cold radioimmunoprecipitation assay buffer [20 mmol/L Tris–HCl (pH 8.0), 100 mmol/L NaCl, 10 % glycerol, 1 % NP40, 0.5 % sodium deoxycholate]. Twenty micrograms of protein were separated in 10 % SDS-PAGE gels and wet transferred to nitrocellulous membrane (GE Healthcare Life Sciences), then blocked for 1 h at room temperature in TBS-T [50 mmol/L Tris–HCl (pH 7.5), 150 mmol/L NaCl, 0.1 % Tween 20] buffer containing 5 % nonfat milk. Membranes were then incubated overnight at 4 °C or 1 h at room temperature with the respective primary antibodies: CIP2A (1:500), pJNK (1:1,000), pATF2 (1:1000), phospho-c-Jun (1:1000) and actin (1:1,000). Anti-mouse or anti-rabbit secondary antibody conjugated to horseradish peroxidase (Santa Cruz Biotechnology) was used to visualize the stained bands with an enhanced chemiluminescence visualization kit (Santa Cruz Biotechnology).

### Production of lentivirus-containing *CIP2A* short hairpin RNA and lentivirus-containing full length CIP2A

Two CIP2A short hairpin RNAs (knock-down), ligated in pLKO.1 vector and pLOX-KIAA1524 (overexpression plasmid containing the full-length CIP2A) were obtained from Open Biosystems. The mature antisense sequences for the two shRNA we used are as following:

- shRNA1: 5’-AAACTTCTCTCAACATACTAGC-3’
- shRNA2: 5’-TTTCTGATTCAACTTGCTGCG-3’

Lentivirus was produced by co-transfection of pLKO.1 control (ligated with scramble sequence) or other pLKO.1-derived vector, or pLOC-KIAA1524 with pMD2.G, and pCMV-VSVG into HEK293T packaging cell lines. The supernatants containing lentvirus of HEK293T were harvested at 36 and 72 h post transfection. Supernatant were pooled, centrifuged to remove cells and then filtered through 0.45 μm low protein binding filter. Cells were plated in monolayer at different densities and infected with lentivirus constructs using 8 ng/mL polybrene. The stable cell lines were selected in the presence of 1 μg/ml puromycin (knock down) or 10ug/ml blastidinin (overexpression) for 2 weeks.

### Screen of phospho-kinase array

Human phospho-kinase array were purchased from R&D system. This array contained 43 antibodies, which were pre-spotted in duplicate on nitrocellulose membranes. Before incubating with cell lysates, membranes were incubated with blocking buffer for 1 h at room temperature. Cell lysates (400 μg protein) were mixed with a cocktail of biotinylated detection antibodies. The mixture was then incubated with the array overnight at 4 °C. After the incubation, membranes were washed three times with washing buffer. Streptavidin-horseradish peroxidase and chemiluminescent detection reagents were subsequently added. Chemiluminescence was detected in darkroom with the same protocol for Western blot. Densitometry was measured with NIH Image J software version 1.45 to quantify the average pixel density/spotted area after normalization to the background controls using plug-in function, protein array. Data are representation of two independent experiments.

### Statistical analysis

For comparisons between two groups, Student *t* test was used. Chi-square analysis was used for categorical outcomes. Both of these tests were performed in PRISM software (GraphPad Software, La Jolla, CA). All tests of statistical significance were two-sided. P values less than 0.01 and 0.05 were considered to be statistically significant.

## Results

### CIP2A elicits autoimmune response in lung cancer patients

**Fig. 1:**
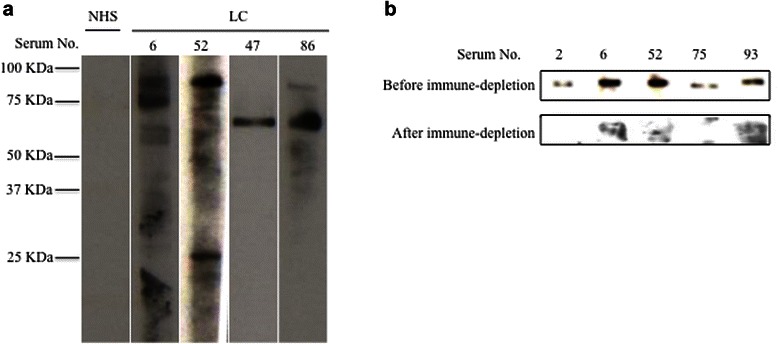
CIP2A elicits autoimmune response in lung cancer patients. **a** Representative Western-blot results of autoantigen screening using human sera. A549 total cell lysates were resolved on 12 % SDS-PAGE gel, and transferred to nitrocellular membrane. The membrane was then cut into stripes to detect the presence of autoantibody in individual human sera. Figure shows lung cancer sera contained autoantibody against a band between 75 kD and 100 kD (serum 6 and serum 52). NHS: normal human sera; LC: lung cancer. **b** Validation of CIP2A autoantigen. Five randomly chosen CIP2A positive sera were used for this validation. Antibodies against CIP2A containing sera were firstly depleted with recombinant GST-CIP2A protein, which was then used for Western-blot for CIP2A fusion protein resolved on SDS-PAGE gel

To identify novel tumor-associated antigens in lung cancer patients, we screened the presence of autoantibodies in 102 sera from lung cancer patients and 82 sera from normal human individuals. As shown in Table 1, 49 out of 102 (48.0 %) lung cancer sera contained autoantibodies while only 5 out of 82 normal human sera were autoantibody positive (6.1 %). Interestingly, among these 49 autoantibody-positive sera, 10 sera contained autoantibodies against a protein migrating around 90 kDa in SDS-PAGE gel, which was not detected in normal human sera (Fig. 1a). The migration pattern of this 90 kDa protein is similar to the previously identified tumor-associated antigen, CIP2A, in gastric cancer, [8].

**Table 1:** Frequency of autoantibody in sera from patients with lung cancer

Type of diseases	No. tested	Frequency (Patients with autoantibody/total patients with disease)
Lung Cancer	102	48.0 % (49/102)**^a^
Normal human sera (NHS)	82	6.1 % (5/82)

^a^ *P* value relative to NHS: * *p* < 0.05; ** *p* < 0.01

To confirm this 90 kDa protein is p90/CIP2A as previously identified, we applied immune-depletion and Western-blot analysis. Five randomly chosen 90 kDa autoantibody positive sera were used as the primary antibody to probe the recombinant CIP2A protein by Western-blot. All of these five sera contained autoantibodies against the CIP2A. The recombinant CIP2A was used to immune-deplete the autoantibodies in each sera, which were used for Western-blot analysis. As shown in Fig. 1b, after the co-incubation of CIP2A recombinant protein with sera, the 90 kDa bands were no longer displayed in all of the five serum sample. Therefore, the protein with 90 kDa band was confirmed to be CIP2A.

To gain a better view of the immune response triggered by CIP2A in lung cancer patients, we used ELISA to measure the frequency of autoantibodies against CIP2A in additional 105 sera from lung cancer patients. Among the 105 samples, 27 sera (25.7 %) contained autoantibodies against CIP2A. While a much lower frequency (4.9 %) of anti-CIP2A positive sera were found in normal human sera (Table 2). The higher percentage of anti-CIP2A positive lung cancer patients than that in normal human suggested CIP2A was more antigenic in lung cancer than in normal human. It also implicated that the expression of CIP2A in lung cancer patients was sensed by the host’s immune system, and thus it can be a potential serum biomarker for lung cancer detection.

**Table 2:** Frequency of autoantibody to CIP2A in sera from patients with lung cancer

Type of diseases	No. tested	Frequency (Patients with anti-CIP2A/total patients with disease)^*a*^
Lung Cancer	105	25.7 %(27/105)**^b^
Normal human sera (NHS)	82	4.9 % (4/82)**^b^

^*a*^ Cutoff value: Mean + 3 SD of NHS

^*b*^*P* value relative to NHS: * *p* < 0.05; ** *p* < 0.01

### CIP2A is overexpressed in lung cancer

**Fig. 2:**
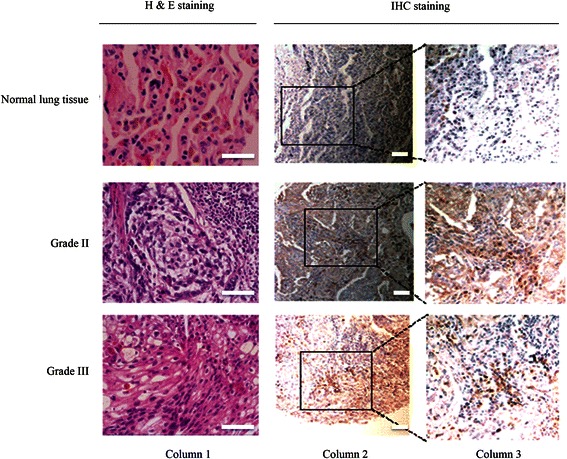
CIP2A expression in normal lung tissue and lung cancer tissue by immunohistochemistry (IHC). Representative IHC images of human lung cancer tissues and normal human lung tissues using monoclonal mouse anti-CIP2A antibody. Column 1 includes H&E stained lung tissues to show the histology of the specimen. Colum 2 showed the IHC results from normal lung tissue, Grade II lung cancer tissue and Grade III lung tissues. Column 3 is the enlarged view of column 2, which is shown as squared region. All scale bars are 50 μm

Although the mechanism of the autoantibody production in cancer is unknown, the elevated expressions of TAAs were frequently observed. Commercially available tumor tissue array was used to examine the expression level of CIP2A in lung cancer tissue specimen. A set of 72 samples of lung cancer tissues, and 63 samples of normal lung tissues were analyzed with immunohistochemistry (IHC) by using monoclonal CIP2A antibody. The CIP2A positive tissue is defined by having a score ranging from 1–6 (Fig. 2). Seven out of 63 normal lung tissues were CIP2A positive, while 61 samples among the 72 lung tumor tissues were CIP2A positive (Table 3). This data suggested that CIP2A was overexpressed in lung cancer. The incidence of lung cancer was correlated with age, especially in people who are 65 years or older. However, the expression level of CIP2A was not statistically associated with the age, and was not correlated with gender, histology, grade or pathological stages (Table 4). As demonstrated by previous study [8, 25], our results also showed cytoplasmic staining of CIP2A (Fig. 2). Taken together, CIP2A is overexpressed in lung cancer tissues compared to normal lung tissues and the resultant overexpression can be considered as a diagnostic factor for clinical lung cancer detection.

**Table 3:** IHC results showing expression level of CIP2A in lung cancer tissue specimens

Tissue status	Total No. of samples	No. of CIP2A positive	*p* value
Normal human tissues	63	7 (11.1 %)	
Lung cancer tissues	72	61 (84.7 %)	<0.001 **^a^

^a^ p value relative to normal human lung tissue. *p < 0.05, **p < 0.01

**Table 4:** CIP2A expression does not correlate with age, gender, stage and histology

Characteristics	CIP2A low expression (score 0–3)	CIP2A high expression (score 4–6)	*p* value
Age			
65<	28 (54.9 %)	23 (45.1 %)	1.00
≥ 65	11 (55 %)	9 (45 %)	
Gender			
Male	25 (47.1 %)	28 (52.9 %)	0.5936
Female	11 (57.9 %)	8 (42.1 %)	
Stage			
I-II	19 (52.8 %)	17 (47.2 %)	0.0935
III-IV	11 (30.6 %)	25 (69.4 %)	
Histology			
Adenocarcinoma	11 (37.9 %)	18 (62.1 %)	0.0951
Non-adenocarcinoma	27 (60 %)	18 (40 %)	

### CIP2A promotes cell proliferation of lung cancer cells

**Fig. 3:**
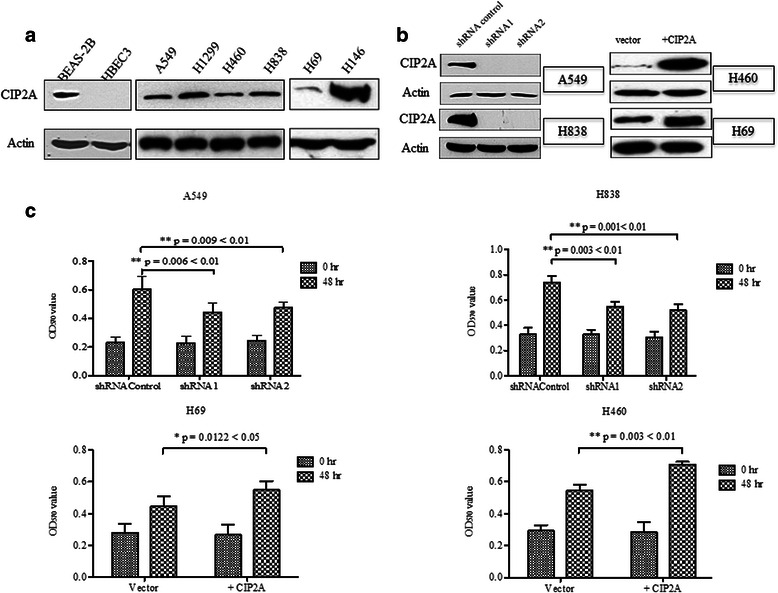
Expression of CIP2A in different lung cancer cell lines and effect of CIP2A in lung cancer cell proliferation. **a** Expression of CIP2A is evaluated in two immortalized lung cell lines, four NSCLC cell lines and two SCLC cell lines using Western-blot analysis. **b** The know down and overexpression effects were examined by Western-blot. In A549 and H838, CIP2A were knock down by two independent shRNAs. In H460 and H69 cell lines, full-length CIP2A were overexpressed by lentiviral transfection. The cell proliferation of these four cell lines was measured at 1st passage. **c** Cell proliferation was performed on four lung cancer cell lines with either knock down (shRNA1 or shRNA2) or overexpression (+CIP2A). For adherent cell lines (A549, H838 and H460), three thousands cells for each cell line were plated triplicate in 96-well plates and MTT solution was added at day 1 and day 3. For suspension cells (H69), cells were resuspended as single cell and 4000 cells were plated triplicate in 96-well plate, and cell proliferation was measured the same as adherent cell. O.D absorbance was read at 570 nm. Statistical significance was done with Students’t test

To examine the role of CIP2A in lung cancer, we first established the expression profile of CIP2A in two immortalized lung cell lines (BEAS-2B and HBEC3), six lung cancer cell lines including four non-small cell lung cancer cell lines (A549, H460, H1299 and H838), and two small cell lung cancer cell lines (H69 and H146). Interestingly, one of the immortalized cell lines, BEAS-2B, did express CIP2A while another cell line HBEC3 was negative of CIP2A expression. In contrast to the immortalized cell lines, all of these six lung cancer cell lines are expressing endogenous CIP2A with distinct expression level (Fig. 3a). H1299 and H146 were expressing higher level of CIP2A than other lung cancer cells while H460 and H69 were expressing lower level of CIP2A.

To assess whether CIP2A could affect lung cancer cell growth, we either depleted CIP2A or increased CIP2A by ectopic expression of the recombinant CIP2A. The expression of CIP2A in different cell lines was evaluated by Western-blot (Fig. 3b). Down-regulation of CIP2A in A549 and H838 cell lines impaired cell proliferation, decreasing to 52.4 and to 47.3 %, respectively (Fig. 3c). In contrast, the overexpression of CIP2A is associated with 23.0 % increase in H460 and 15.6 % in H69 cell lines. Therefor, loss-of-function and gain-of-function studies suggested that CIP2A promote cell proliferation of lung cancer cells.

### CIP2A positively enhances c-Jun N-terminal kinase (JNK) pathways in lung cancer cells

**Fig. 4:**
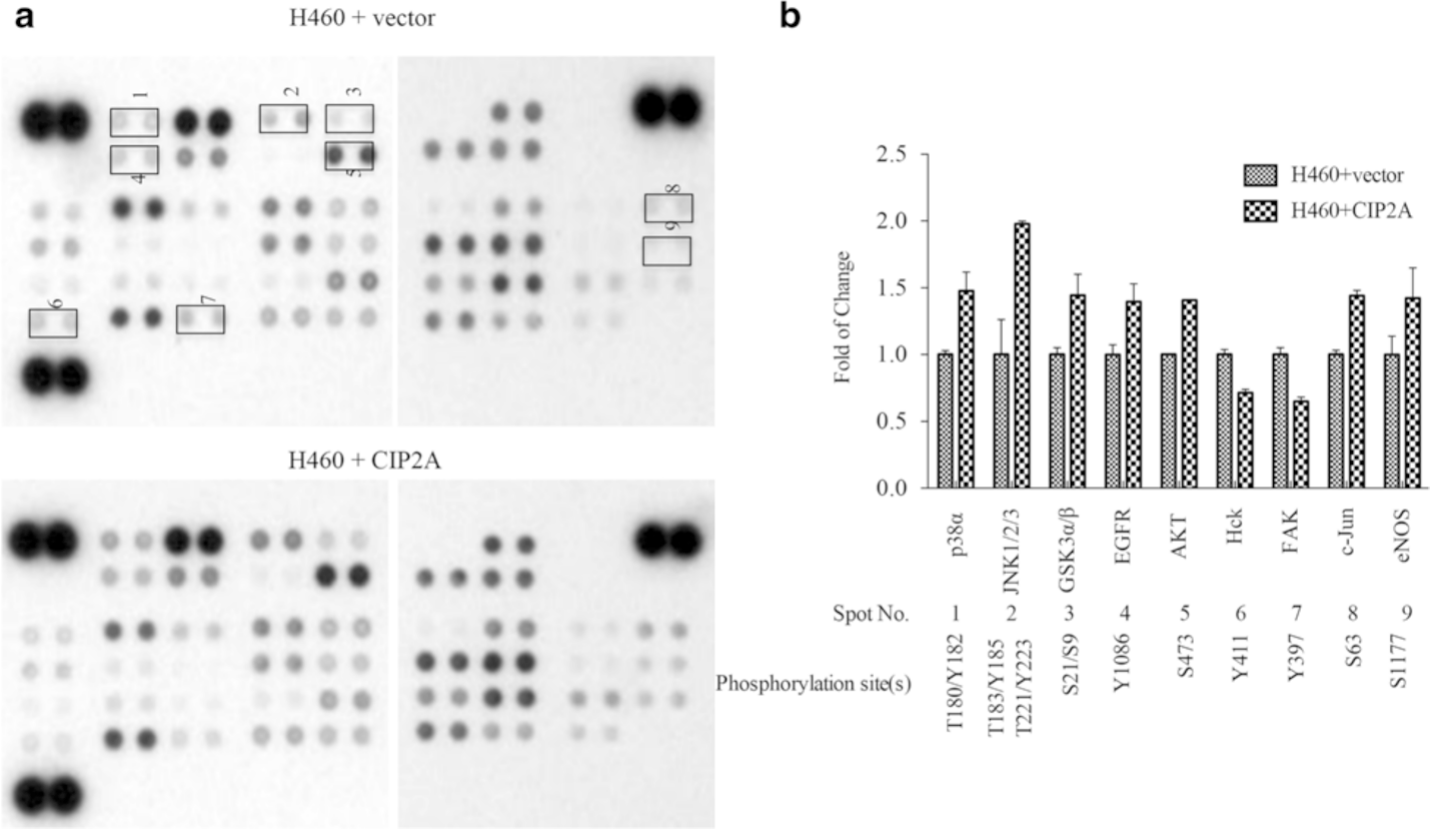
Analysis of CIP2A on phosphorylation of 43 key signaling molecules using phospho-array in H460 cell line. **a** Phospho-array analysis of the phosphorylation of key molecules in signaling pathways using Human phospho kinase array. Highlighted spots are molecules showing fold of change > 1.4 between H460 cell line and H460 overexpressing CIP2A cell lines. Fold of change is measured by densitometry. **b** Differentially phosphorylated molecules between H460 + vector and H460 + CIP2A with information regarding their fold of changes and phosphorylation site(s). JNK, c-Jun activated kinase (JNK); GSK α/β, glycogen synthasase kinase α/β; EGFR, epidermal growth factor receptor; Hck, hemopoietic cell kinase; FAK, focal adhesion kinase (FAK)

To identify proteins involving in CIP2A-mediated cell proliferation, we performed human phospho-kinase array, which include 43 representing phosphorylation events of kinases in cell in response to environmental change, to identify the possible molecules associated with the expression change of CIP2A. Total proteins extracted from H460 cells transfected with vector control (H460+ vector) or from H460 overexpressing CIP2A (H460 + CIP2A) were used as antigen to react with the phospho-antibody array, which was subsequently detected with phospho-antibody cocktails (Fig. 4a). Several molecules with detectable difference in phosphorylation states between the control and overexpression groups (with >1.4 fold change) were listed in Fig. 4b. Of notice, the most altered phosphorylation molecule was pan-JNK, which increased as much as 1.9 fold upon the increased expression of CIP2A.

### CIP2A enhances MKK4/MKK7-JNK-c-Jun pathway to promote cell proliferation

**Fig. 5:**
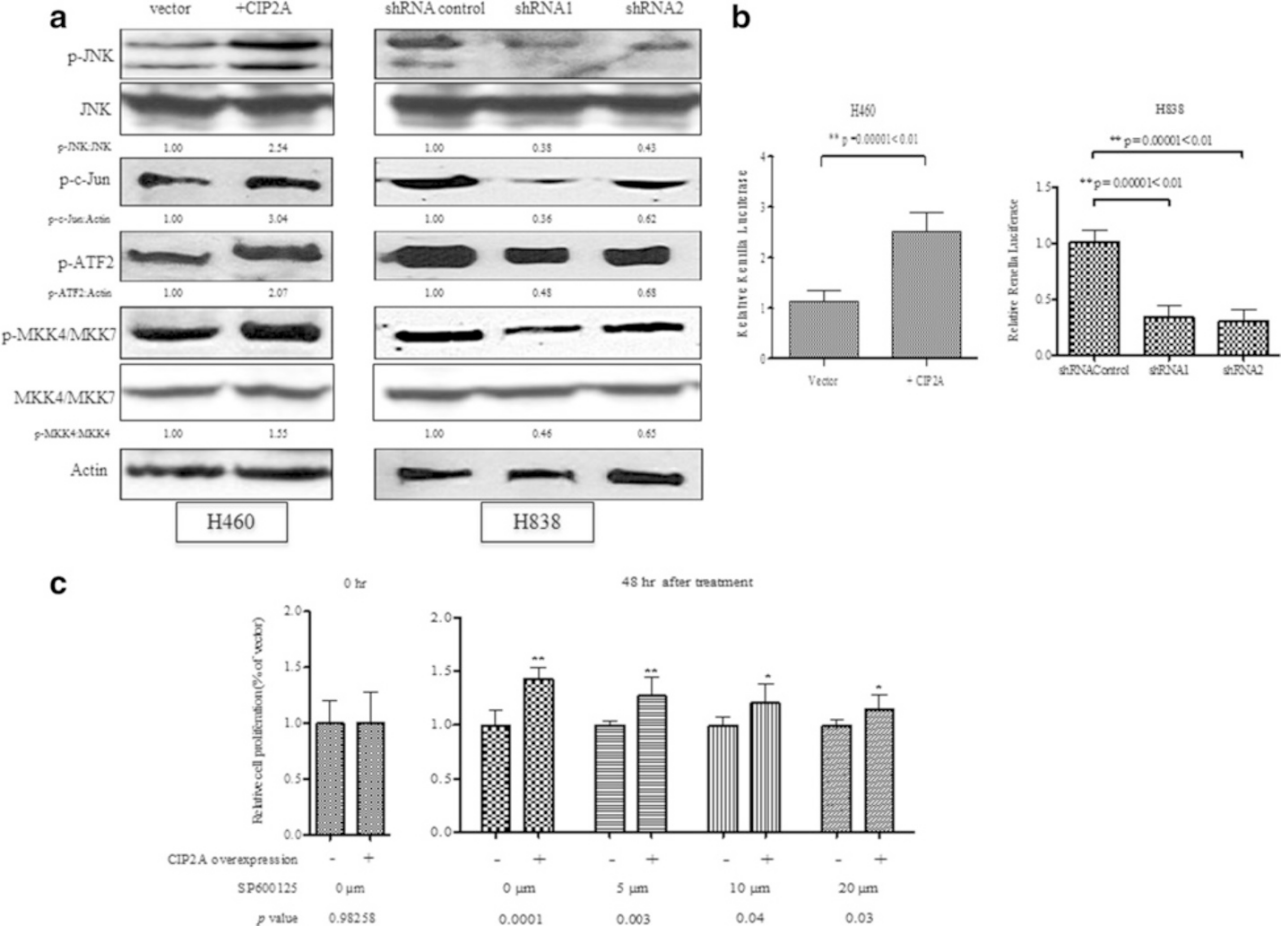
CIP2A modulate JNK signaling pathway and transcriptional activity. **a** Western-blots showed the phosphorylation changes of MKK4/7, JNK, c-Jun and ATF2 in association with the expression change of CIP2A in H460 and H838. Densitometry analysis performed in Image J showed the folds of phosphorylation change. The phosphorylation changes were normalized to non-phosphorylated form which was normalized with actin. **b** The luciferase activity of the downstream target of JNK, AP-1, was measured in H460 and H838 with overexpression or knock down of CIP2A. **c** Cell proliferation assay of H460 and H460 overexpressing CIP2A cell lines in the presence or absence of JNK inhibitor, SP10025

JNK belongs to the family of mitogen-activated protein family (MAPK), which responds to stress stimuli like UV radiation, cytokines, heat shock and osmotic shock. Activated JNK is associated with increased cell proliferation, cell migration and cell invasion in non-small cell lung cancer. Therefore, it is possible that CIP2A may regulate lung cancer progression in a JNK signaling-dependent manner. To validate our finding that CIP2A regulates JNK phosphorylation in lung cancer cells, we treated the cells with EGF to activate the growth factor-induced JNK activation for 15 mins (Fig. 5a). Consistent with tphospho-kinase array, the phosphorylation status of JNK is correlated with the expression level of CIP2A. The overexpression of CIP2A in H460 cell line greatly enhanced the phosphorylation of JNK. While the knockdown of CIP2A in H838 lead to the reduced phosphorylation of JNK to 2.5 fold. Furthermore, the phosphorylation of downstream substrates of JNK, c-Jun and ATF2 were also altered along with the phosphorylation of JNK (Fig. 5a). In addition, the phosphorylation of upstream kinases of JNK, MKK4/MKK7, was also reduced upon CIP2A knock down. However, many kinases are upstream of MKK4/MKK7. It is hard to determine which specific kinase is affected due to the expression level change of CIP2A. Therefore, CIP2A may be a modulator of JNK pathway by targeting upstream kinases of JNK.

To test whether the transcriptional activity of the downstream molecule of JNK, we measured the AP-1 transcription activity using luciferase reporter assay. The overexpression of CIP2A increased the AP-1 transcriptional activity to at least 2.3 folds while the depletion CIP2A in H838 reduced of AP-1 transcriptional activity around 30–35 % of the control group (Fig. 5b).

JNK pathway is frequently activated in lung cancer during cell transformation and lung cancer cell progression. Therefore, it is possible that CIP2A may promote cell proliferation in a JNK-dependent manner. We treated CIP2A-overexpressing H460 with different concentrations of JNK inhibitor, SP610025. After 48 h, cell proliferation was measured with MTT assay. As shown in Fig. 5c, in the DMSO (0 μm) treated group, the cell proliferation of CIP2A overexpression cell line was 41.6 % higher than the control group. Upon JNK inhibitor treatment, the cell proliferation of CIP2A overexpressing cell lines was decreased to 27 % when treated with 5 μm inhibitor. However, the treatment of CIP2A overexpressing cell line with higher concentration of 10 and 20 μm did not significantly reduce cell proliferation, which is 20 % of the control cell line (Fig. 5c). This indicated that inhibition of JNK pathway only partially inhibit CIP2A-induced cell proliferation.

## Discussion

The relationship between the immune system and tumorigenesis is not only intrinsically important, but also fundamental to the design of future cancer therapies. The fact that proteins involve in tumorigenesis and cancer progression can be sensed by host’s immune system promoted researchers to profile proteins with immunogenic ability during the onset and progression of diseases [26]. Our lab has well-established platforms to identify this type of proteins in cancer patients by either cDNA expression library screening or proteomic approaches [27]. A series of such proteins had been identified like p62/IGF2BP2, p90/CIP2A, Hsp60 and Hsp70 [8, 28, 29]. In this study, we not only found that autoantibodies against CIP2A had very high frequency (25.7 vs 4.9 % in normal) in the sera from lung cancer patients but also the overexpression of CIP2A in more than 60 % lung cancer tissues. Of notice, CIP2A was previously shown as a developmentally regulated protein, whose expression was restricted to embryonic stages but was silenced in normal adult tissues [8]. Therefore, both of these evidences supported that aberrant CIP2A expression at early stage of cancer would trigger immune response. Although CIP2A is not secreted into the extracellular space nor present as membrane-bound form, it is possible that immune response was initiated when nascent cancer cell was recognized by the immune system and lysed to release intracellular proteins, which induce autoantibody generation. The presence of autoantibody against CIP2A in sera from lung cancer patients also suggested that CIP2A was activated at early stage of lung cancer initiation. Furthermore, the expression of CIP2A in pre-malignant stage contributed to tumor formation [25]. And studies from other research groups reported that CIP2A overexpression were found in more than 70 % of other types of cancer patients [9–16] . Taken together, the temporal expression pattern makes CIP2A as an ideal candidate for early cancer diagnosis. However, CIP2A may not be used as an individual diagnostic factor or as biomarker for certain types of cancer cancer; instead it can serve as a valuable factor when combining with other clinically established biomarkers for specific cancer detection.

During our evaluation of CIP2A expression in different lung cancer cell lines, one interesting observation is the expression of CIP2A in immortalized cell line BEAS-2B but not in another immortalized cell line HBEC3. Although many studies have demonstrated the overexpression of CIP2A in cancer cell lines, few studies had examined the expression in immortalized cell line. The expression difference between the pre-malignant cell lines may be due to the different genetic background. The BEAS-2B was immortalized with chimera viral protein while HBEC3 cell line was immortalized by inactivating only an individual protein, p16. The viral chimera protein may activate the transcription CIP2A but not by p16-mediated pathway. This is also consistent with the study that the expression of CIP2A does not statistically correlated with the expression of p53. In addition, the expression of CIP2A in immortalized cell line is an alternative evidence that CIP2A can be present in pre-malignant tissue as reported previously that CIP2A was present in pre-malignant tissue in the mouse studies as well as the in the patients’ tissues of head and neck cancer [25]. Taken together with the results from other groups, our data implied CIP2A might be a prerequisite to cellular transformation.

Although abundant evidences had shown that CIP2A is overexpressed in many types of cancer, the role of this protein in cancer progression is still unknown. In our study, we found that CIP2A overexpression is correlated with increased phosphorylation of JNK. JNK bears both of tumor-promoting and tumor-suppressive function depending on the types and genetic contexts of the tumor cells [30]. Although growth factors like EGF can activate the tumor-promoting function and the environmental stress like UV can activate the cell death pathway, the molecules in making this decision is nevertheless not determined. Our finding that CIP2A enhanced the activity of JNK signaling pathway and lead to the increased cell proliferation suggested CIP2A can be a regulator in determining the substrate selection and prime the cell fate for proliferation rather than apoptosis or necrosis. However, one challenging problem was to fish out the proteins targeted by CIP2A. The rationale behind this was based on the PP2A inhibitive ability of CIP2A. We have searched literature about the PP2A targets of JNK signaling pathways, it involves more than ten proteins upstream of MKK4/MKK7 with each has multiple phosphorylation events. We therefore failed to find the CIP2A targets in JNK signaling pathway due to the technique difficulties. Interestingly, another two identified PP2A inhibitors, I2PP2A and small t antigen have similar roles to CIP2A in promoting JNK phosphorylation and AP-1 transcriptional activity [31]. However, the exact mechanism of how these PP2A inhibitors, including CIP2A, are able to modulate the JNK phosphorylation signaling in a direct or an indirect way is still unknown. A systematic investigation of the global phosphorylation change might be employed to identify the CIP2A targets in JNK signaling pathway.

Our phospho-antibody array has found the phosphorylation change of several other signaling molecules like AKT, GSK3beta and p38. The role of CIP2A in promoting AKT phosphorylation in a stress-dependent and growth-factor-dependent way has been well established. CIP2A in promoting AKT-mediated signaling through the effect on AKT-associated PP2A phosphatase activity has been confirmed in hepatocellular carcinoma, breast cancer and lung cancer cell lines. For GSK3beta and p38, we actually did not find overexpression or knockdown of CIP2A has direct effect on the phosphorylation of these two proteins by Western blot. And our results that CIP2A does not affect the protein level of beta-catenin indirectly supported that CIP2A does not regulate the phosphorylation of GSK3beta. Although MKK4/MKK7 activates both JNK and p38, we here only showed CIP2A regulates JNK phosphorylation.

Our study of CIP2A-mediated cell proliferation partially through priming JNK signaling for cell proliferation rather than cell apoptosis expanded the understanding of oncogenic function of CIP2A in cancer progression. An important aspect in our study is that JNK is only partially contribute to CIP2A-mediated cell proliferation indicating CIP2A has multi-targets, which synergistically to promote cell proliferation. The previous findings that CIP2A physically interacted with c-myc to increase stability, and bind with Plk1 and NEK2 to extend cell division are the possible reasons that inhibition of JNK pathway only partially rescued CIP2A-associated cell proliferation [19, 20, 25]. Moreover, our recent evidences that CIP2A regulated cell proliferation through AKT/PKB signaling pathway, and promoting cancer metabolism enriched the network being played by CIP2A [32, 33]. However, how these CIP2A targeted molecules and signaling pathways are united together in cancer progression require extensive systematic studies.

## Conclusion

In this study, we mainly demonstrated that CIP2A is overexpressed in lung cancer and mount immune response in a high percentage of lung cancer patients. And the overexpressed CIP2A in lung cancer could enhance JNK signaling through the phosphorylation of MKK4/MKK7-JNK-c-Jun pathway. Our data could have two impacts, CIP2A can be a candidate biomarker for cancer diagnosis and CIP2A has novel function in JNK signaling pathway in cancer progression.

## Abbreviations

TAA: Tumor-associated antigen
PP2A: Protein phosphatase 2A
CIP2A: Cancerous inhibitor of PP2A
JNK: c-Jun activated kinase
MKK4/7: Mitogen-activated kinase 4/7
